# Gross transcriptomic analysis of *Pseudomonas putida* for diagnosing environmental shifts

**DOI:** 10.1111/1751-7915.13404

**Published:** 2019-04-07

**Authors:** Ángeles Hueso‐Gil, Belén Calles, George A. O'Toole, Víctor de Lorenzo

**Affiliations:** ^1^ Systems Biology Program Centro Nacional de Biotecnología‐CSIC Campus de Cantoblanco Madrid 28049 Spain; ^2^ Department of Microbiology and Immunology Geisel School of Medicine at Dartmouth Hanover NH 03755 USA

## Abstract

The biological regime of *Pseudomonas putida* (and any other bacterium) under given environmental conditions results from the hierarchical expression of sets of genes that become turned on and off in response to one or more physicochemical signals. In some cases, such signals are clearly defined, but in many others, cells are exposed to a whole variety of ill‐defined inputs that occur simultaneously. Transcriptomic analyses of bacteria passed from a reference condition to a complex niche can thus expose both the type of signals that they experience during the transition and the functions involved in adaptation to the new scenario. In this article, we describe a complete protocol for generation of transcriptomes aimed at monitoring the physiological shift of *P. putida* between two divergent settings using as a simple case study the change between homogeneous, planktonic lifestyle in a liquid medium and growth on the surface of an agar plate. To this end, RNA was collected from *P. putida* KT2440 cells at various times after growth in either condition, and the genome‐wide transcriptional outputs were analysed. While the role of individual genes needs to be verified on a case‐by‐case basis, a gross inspection of the resulting profiles suggested cells that are cultured on solid media consistently had a higher translational and metabolic activity, stopped production of flagella and were conspicuously exposed to a strong oxidative stress. The herein described methodology is generally applicable to other circumstances for diagnosing lifestyle determinants of interest.

## Graphical abstract

This article proposes an standardized workflow for generation and analyses of transcriptomes of *Pseudomonas putida*.

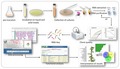

## Introduction

Before the onset of genomic technologies, the way to inspect complex environmental adaptations of microorganisms largely relied – whenever possible – on generation of mutant libraries followed by phenotypic characterization. Today, the most popular approach to the same end typically starts with generation of a differential transcriptome for comparing genome‐wide gene expression patterns in condition A vs. condition B. While the platforms and technologies available to this end have evolved over the years, for example, from DNA arrays to deep sequencing of transcripts (RNA‐Seq), the results deliver a list of genes that go up or down depending on the specific scenarios and their differences (Wang *et al*., 2009). Such profiles not only expose global physiological responses, but also pinpoint the roles of distinct genes that can then be separately studied. Without surprise, *Pseudomonas putida* has not been alien to such technical and conceptual developments and a suite of transcriptomes of this bacterium have been published in recent years with different methods for inspecting the responses of this microorganism to different physicochemical settings (Yuste *et al*., 2006; Kim *et al*., 2013, 2016; La Rosa *et al*., 2015; Bojanovic *et al*., 2017; Molina‐Santiago *et al*., 2017). However, such experiments were run by different laboratories with diverse RNA extraction and preparation methods, and with different data analysis and representation tools, and thus make comparisons challenging. In this context, the protocols below are an attempt to set a standardized workflow for generation of complete and reliable transcriptomes of *Pseudomonas putida* aimed at identifying genes and functions involved in lifestyle transitions. To this end, we have chosen two simple conditions that are habitual in the laboratory but involve completely different sets of circumstances: growth in liquid medium in a rotating tube and growth on the agar surface of a Petri dish. The nutritional conditions in either case are the same (see below), but the rest of the physicochemical settings are very different. Comparison of the two should thus shed some light on what is being sensed as a new niche and also what functions are involved in the transition and thriving in either place. Obviously, one major change is the shift between a basically homogeneous, liquid and water‐saturated environment towards one that is semi‐solid, exposed to aerial desiccation and with nutrients available only from the lower, stiff substratum layer. Genes are thus anticipated to appear involved in surface sensing and changing from a planktonic to a sessile lifestyle (Schembri *et al*., 2003; Oggioni *et al*., 2006; Dotsch *et al*., 2012), but many others could be expected as well. It is remarkable that despite intensive studies of *P. putida*, many questions on the physiology of this bacterium on an agar plate remain unanswered. To shed light on these uncertainties, we simply compared the transcriptomes generated with RNA extracted from *P. putida* KT2440 grown in liquid M9 mineral medium supplemented with citrate as sole carbon source, either on tubes on rotation or on Petri plates made with the same components but containing 1.5% bacteriological agar. As shown below, the results provided hints about the functions and pathways involved in adaptation from liquid to solid media, as well as indications of the type of environmental conditions experienced by bacteria on an agar surface.

## Protocols

### Culture incubation conditions

For this experiment, the strain of *Pseudomonas putida* KT2440 was used. Therefore, the required temperature for cultivation was 30°C but this can be changed depending on the microorganism or strain under study.

- Place an inoculum from the glycerol stock in a 15 ml tube with 5 ml of M9 minimal medium supplemented with 0.2% (v/w) citrate and rotate overnight (O/N) with good aeration at 30°C.
- After that incubation period, inoculate 5 ml M9 citrate 0.2% (v/w) liquid medium in the same type of tube with 100 μl of the O/N culture and place it in a rotator wheel at 30°C.
- Synchronized with step 2, take another 100 μl of the O/N culture and spread it over 25 ml plates of M9 0.2% citrate solidified with 1.5% (v/w) agar. Spread the culture all over the surface using a sterile glass handle. Place plates at 30°C.

### RNA extraction

For the RNA extraction and the following DNA degradation, it is important to utilize clean material that is only for this use. To this end, tips (recommendable with filter) should be fresh or double‐autoclaved. Pipettes and bench should be cleaned thoroughly with ethanol, and brand‐new gloves are necessary during the whole process. Avoid touching untreated surfaces during the application of the methods. The procedure asks for having handy ^a^ RNeasy Kit (Qiagen, Venlo, Netherlands). Buffer RLT^1^ included in the kit should be mixed fresh with 1% β‐mercaptoethanol right before use. Also, a fresh 3 mg ml^−1^ lysozyme solution in 10 mM Tris‐HCl (pH = 8.0) should be ready at the time RNA extraction starts.

- After the required incubation period, collect cells from the cultures in liquid and solid media. In the case of liquid media, the whole 5 ml has to be centrifuged in order to obtain enough RNA using a table‐top centrifuge at 4°C and 10 000 *g* during 5 min. It can be distributed in 1–2 ml aliquots for the centrifugation.
- Gather all the bacteria from solid cultures using a sterile plastic scraper and 5 ml of PBS buffer 1× (137 mM NaCl, 2.7 mM KCl, 10 mM Na_2_HPO_4_, 1.8 mM KH_2_PO_4_, pH 7.4) pre‐chilled at 4°C. Distribute PBS re‐suspension in 1 ml aliquots (it is important to not overload the lysis buffer) into twice‐autoclaved 1.5 ml tubes. Centrifuge as in step 1.
- Quickly place pellets at −80°C for 10 min.
- Thaw frozen pellets at room temperature for 5 min maximum.
- Resuspend tube content in 100 μl of 3 mg ml^−1^ lysozyme solution. Keep at room temperature for 3 min exactly.
- Add 350 μl of β‐mercaptoethanol/buffer RLT and mix thoroughly by vortexing.
- Add 250 μl of 100% ethanol to the lysate and mix by pipetting.
- Add all the 700 μl to a mini‐spin silica membrane column, put it inside a RNase‐free 1.5 ml tube and spin down for 15 s at 16 000 *g*.
- Discard the collection tube and insert the column into a new one. Put in 700 μl of RW1 washing buffer of the RNeasy kit (see footnote 1) and spin down as in step 8.
- Transfer the column again to a new collection tube and discard the previous tube. Add 500 μl of buffer RPE provided by the kit (see footnote 1) and spin the same way as previously. Discard flow through.
- Add 500 μl of Buffer RPE and centrifuge 2 min at maximum speed.
- Transfer the column to a new collection tube and centrifuge for 1 min at maximum speed.
- Move column to a double‐autoclaved 1.5 ml tube and elute with RNase‐free water. Centrifuge for 1 min at maximum speed.
- Repeat step 13 in order to have a total elution volume of 60 μl.

### DNA removal

We suggest to employ the DNA‐free kit (Ambion) (Invitrogen, Carlsbad, CA, USA) for the DNA purification from the samples as follows.

- Add 6.5 μl of 10× Buffer to the 60 μl RNA elution from previous step.
- Add 1 μl of DNase I to each tube and mix gently by pipetting (not vortexing).
- Incubate at 37°C for 60 min.
- Before use, DNase inactivation buffer should be thawed slowly during the incubation of step 3.
- Add 7 μl of inactivation buffer and leave the tube for 2 min at room temperature, flicking it every 30 s.
- Centrifuge at 10 000* g* during 1.5 min.
- Transfer supernatant to a clean RNase‐free 1.5 ml tube very carefully.

### Re‐purification

- Adjust the sample volume to 100 μl with RNase‐free water.
- Add 350 μl of β‐mercaptoethanol/RLT buffer and mix using the pipette.
- Add 250 μl of ethanol and mix by pipetting again.
- Transfer the 700 μl to a mini‐spin silica membrane column and centrifuge for 15 s at 16 000 *g*. Discard the flow through and place the column in a new collection tube.
- Add 500 μl of the RPE buffer provided by the kit and spin as in step 4. Place the column in a new collection tube as previously.
- Repeat the previous step but centrifuging for 2 min at 16 000 *g*.
- Place the column in a 1.5 ml tube and add 50 μl of RNase‐free water.
- Centrifuge for 1 min at the same speed.

### Second DNase treatment and RNA quality control

Repeat section 3 (DNA removal) taking special care in transferring only the supernatant to a new RNase‐free 1.5 μl tube and not the pellet resulting from the last centrifugation. The RNA concentration and its purity can be measured with a NanoDrop machine (Eppendorf, Hamburg, Germany). In addition, we recommend to perform a qRT‐PCR analysis of all the samples with some known primers using *P. putida* genomic DNA as a positive control in order to ensure that RNA preparations give no amplification (sometimes the DNA is not completely degraded). Should this happen, DNA elimination step should be done once more. Primers usable for this control included in our case sequences targeting inside the polysaccharide transporter PP_3132 in *pea* operon and PP_1795 in *peb* operon of *P. putida* (Table S1).

### RNA‐Seq

Actual deep sequencing of the high‐quality RNA samples prepared as explained above is typically outsourced in a core facility of the institution where experiments are done or arranged with a commercial sequencing company. For the data discussed below, the RNA samples were processed for sequencing in the transcriptome analysis services of the Helmholtz Centre for Infection Research (HZI) in Braunschweig, Germany.

### RNA‐Seq data processing

For the processing of raw data, we adopted a generally utilized methodology and recommend some bioinformatic programmes for every step. Note, however, that the same tasks can be accomplished using other existing software. Prior to the analyses, proper, raw reads in FASTQ format need to be checked for its quality with a suitable tool, for example FastQC (Brown *et al*., 2017). After that, procedure goes as follows:

- Each sample has to have its single‐end reads aligned against the reference genome, in our case, against *Pseudomonas putida* KT2440 genome (assembly: GCA_000007565.2 ASM756v2). This can be done with Bowtie 2 (Langmead *et al*., 2009; Langmead and Salzberg, 2012).
- SAM alignment files are frequently converted to BAM files, sorted and indexed using SAMtools (Li *et al*., 2009; Li, 2011).
- In order to visualize the files, Integrative Genomics Viewer (IGV) is an appropriate programme (Robinson *et al*., 2011; Thorvaldsdottir *et al*., 2013).
- HT‐seq (Pyl *et al*., 2014) set in *union* mode is a valuable software to count the number of reads per gene and quantify expression levels. Gene coordinates are necessary for this step; they can be downloaded in gff format from the suitable webpage. For our analyses, we used https://www.ncbi.nlm.nih.gov/genome/?term=Pseudomonas%20putida%20KT2440
- A later normalization of read counts and differential gene expression is required. For that objective, DESeq2 (Love *et al*., 2014) works correctly.
- Preparation of tables should include several columns of informative data, such as raw and normalized counts, logFoldChanges, *P*‐values, *P*‐adjusted values and functional description for each gene in order to facilitate its transfer and visualization using Fiesta viewer (Oliveros, 2007a).

The comparisons were set taking the results of the liquid culture as the reference, so the positive fold‐change values are interpreted as an upregulation on solid media, while negative fold‐change values represent downregulation on agar plates. For further details about this section and additional features on the above‐mentioned programmes, see Oliveros ([Link]). Genes undergoing significant variations can be selected using the fiesta Viewer v.1.0 software (Oliveros, 2007a,2007b), setting the parameters of *P*‐adjusted value < 0.001 and fold change of < −2/> 2. However, it can be changed whenever required, and < 0.01 and < 0.05 are acceptable options, too.

### Functional analyses

The list of selected genes with a differential regulation has to go through a last processing step. To this end, we suggest to upload the resulting lists into the david V6.8 platform (Huang da *et al*., 2009a,2009b). david software was used in order to organize the list of genes according to their functions and their relevance to this study. For this purpose, EASE Score, also known as modified Fisher's exact *P*‐value (Hosack *et al*., 2003), is a parameter that considers the number of differentially expressed genes from an specific functional group and it compares these genes not only to the total number in the results, but also to the number of annotations with that specific role present in the genome. Therefore, the EASE Score is a good statistic to visualize how important a group of genes is for the experimental conditions tested: a smaller EASE Score means a higher enrichment of the annotation, so to make a more intuitive representation of the statistic, the –log(EASE Score) is usually calculated and plotted. In that way, a higher value of the –log(EASE Score) also means a higher enrichment of the function. It has to be noted that this statistic does not consider whether the genes are negatively or positively regulated, nor the fold‐change value. So, when more detailed information is needed, fold‐change values should be consulted in the lists. For more specific records on distinct genes, the *Pseudomonas* Genome DB (Winsor *et al*., 2016) is a most useful resource.

Although there are multiple ways to group data using david software, we propose a functional annotation chart setting with only four parameters: three types of Gene Ontology (GO) terms (GOTERM_BP_DIRECT, GOTERM_CC_DIRECT and GOTERM_MF_DIRECT) and one pathway (KEGG_PATHWAY; Huang da *et al*., 2009a,2009b). DAVID information has to be downloaded and can be plotted using Excel and GraphPad Prism 6. Another useful online software to produce Venn diagrams is venny 2.1 (Oliveros, 2007b).

## Application example

### Gross transcriptome results

**Figure 1:**
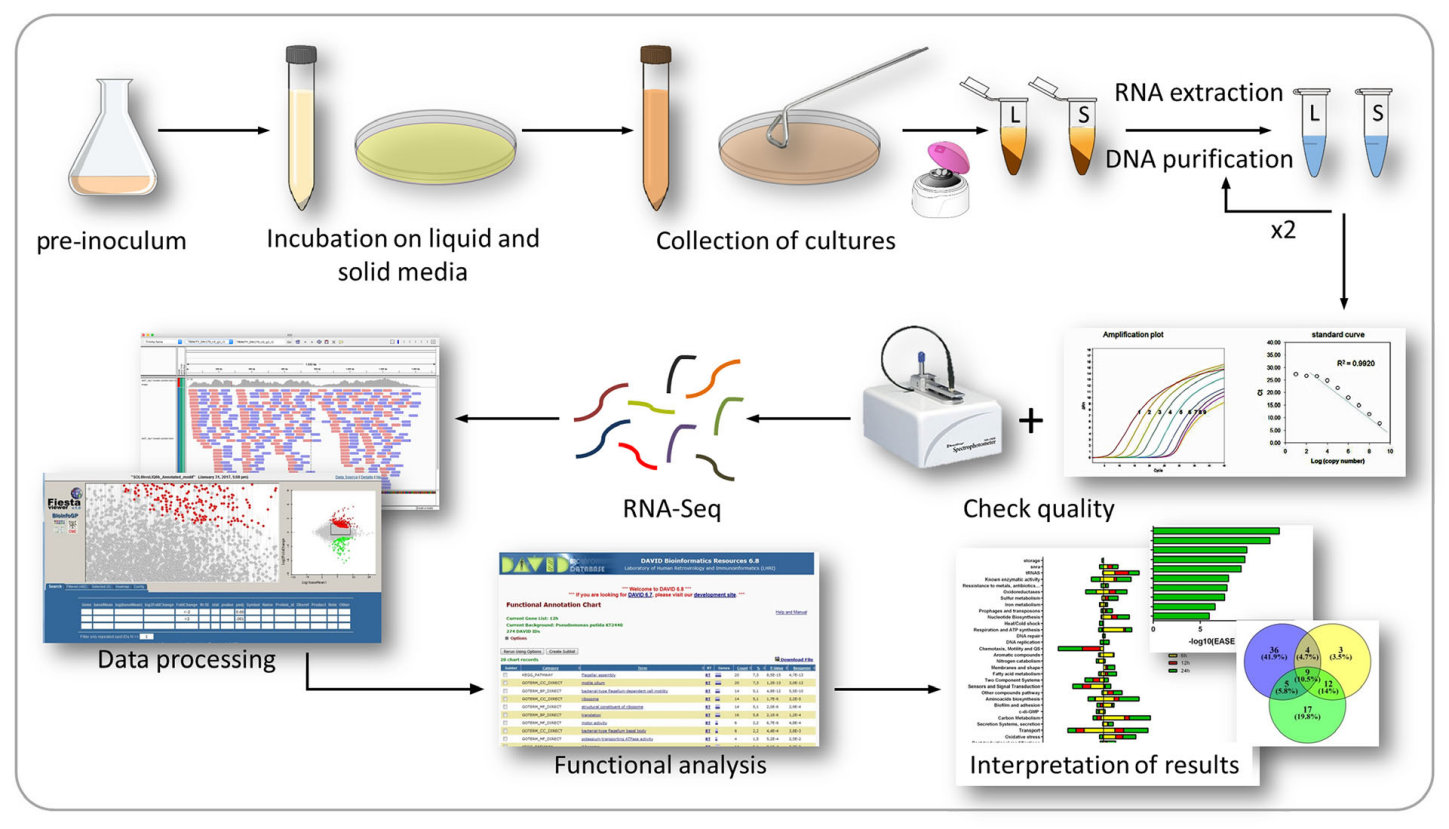
Workflow of the general procedure explained in these protocols. The main wet and *in silico* steps are summarized in the cartoon. The biological materials are retrieved from cultures of the same *P. putida* cells in either liquid M9‐citrate medium or plates with the same composition but solidified with 1.5% agarose.

The workflow of the protocol described above is sketched in Fig. 1. Bacteria grown on solid and liquid media were harvested at three different time points in order to have an illustration of three stages of the bacterial physiological state: 6 h as a representation of exponential phase, 12 h as the time for the early stationary phase and 24 h as a time for late exponential phase. Two biological replicates were run per condition.

**Figure 2:**
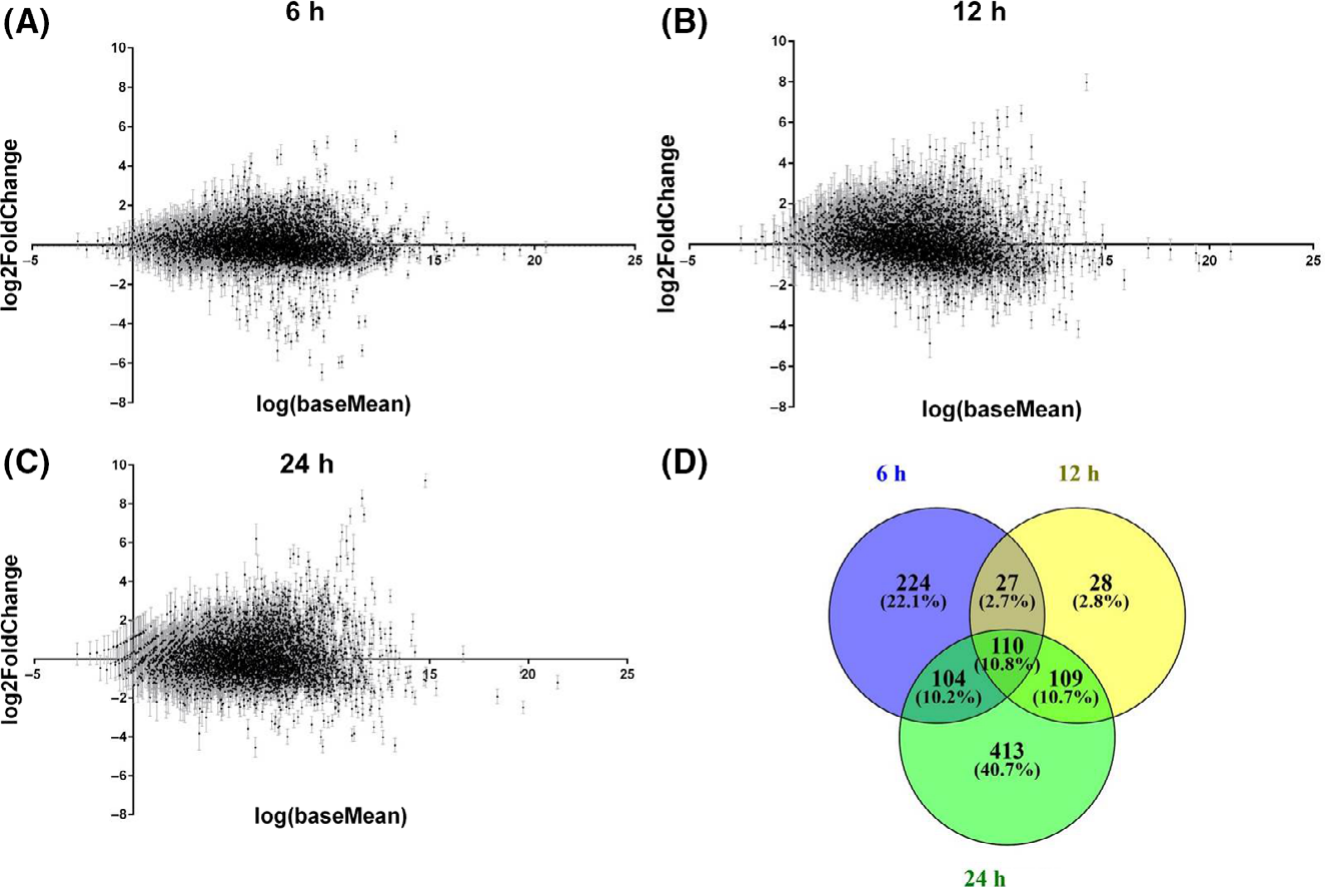
Dot plot representation of the RNA‐Seq results. The data compare the transcriptomes of *Pseudomonas putida* KT2440 cultured in solid media vs. cultured in liquid media at (A) 6 h, (B) 12 h and (C) 24 h. Each point represents a different gene with its error bar, and the scattered points at both sides of the *x*‐axis show the up‐ or downregulation of them comparing both solid and liquid way of culture. (D) Venn diagram for the genes differentially expressed at the three different time points.

The gross results of the experiments are summarized in Fig. 2. The number of sequences that resulted differentially expressed with a *P*‐adjusted value of 0.001 and fold change of <−2/> 2, which were applied as selection parameters, was of 465 genes when liquid and solid cultures were stopped at 6 h, 273 genes at 12 h and 736 genes at 24 h (full lists are attached in the Tables S2–S7). One remarkable result was the lack of differential transcription of *rpoS* (PP_1623) at comparable times in liquid and solid media. Since this gene codes for the stationary‐phase sigma factor, the score indicated that the growth stage is comparable in either condition and thus that the differences ought to be due to other physicochemical circumstances. In addition, *rpoD* (PP_0387), the RNA polymerase σ^70^ factor, sometimes used as a negative control for qRT‐PCRs, remained also constant in all the comparisons.

### Transcription and translation activities

**Figure 3:**
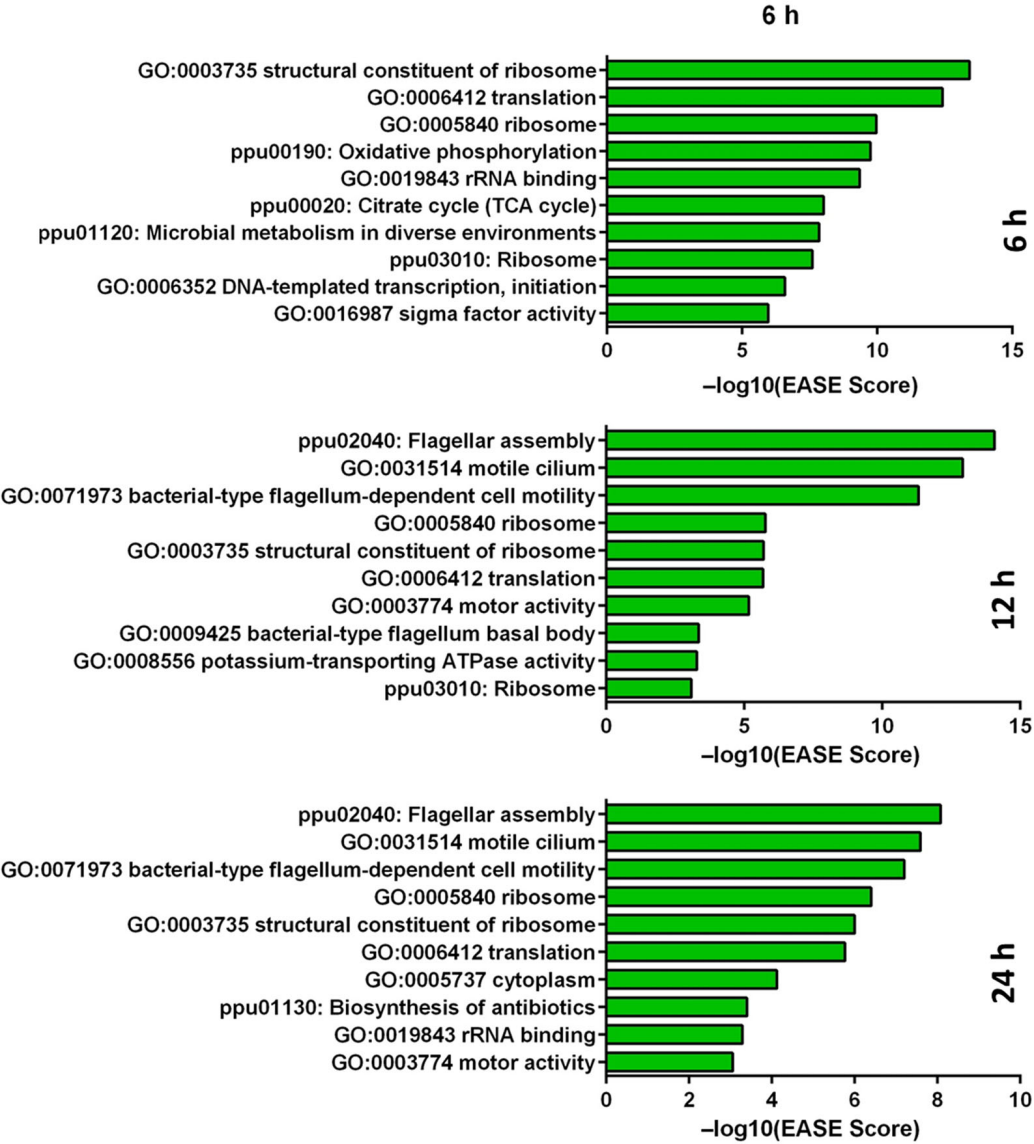
Representation of the 10 most relevant annotations. Functions/genes of interest appear as GO terms (Biological Process, Molecular Function and Cellular Component) and KEGG pathways (classified as ppu+number). Its –log(EASE Scores) assigned values are plotted according to their value in the transcriptomes at 6, 12 and 24 h once data have been processed using the david software.

As shown in Fig. 3 and Table 1, translation seems the most differentially regulated function at 6 h of incubation. There was a conspicuous appearance of genes related to ribosome subunits and translation itself with some of the lower EASE Scores and, therefore, a high value of its negative logarithm number. This general upregulation is connected to the observation that some genes involved in transcription are also positively regulated, including sigma factors. These results suggest that bacteria cultured on solid media have a higher metabolic activity and more proteins are produced as a consequence. These are probably enzymes and structural proteins that allow bacteria to face the characteristic conditions of solid surfaces, as, for example, special requirements to internalize nutrients, production of extracellular polymeric substances (EPS), resistance to stresses (such as desiccation, oxidation) and other functions which demand a higher level of transcription and translation. This upregulation is generally maintained through 12 and 24 h, although there were notable exceptions that included, for example, regulators of iron metabolism, for example the sigma factor σ^19^ and the *pvdI* gene, which were clearly downregulated at 6 h (although differences tend to level off with time). The same was true for the regulators of flagellar production and motility such as *fliA* or *fleS,* which become remarkably undertranscribed at later times (see Tables S1–S7).

**Table 1:**
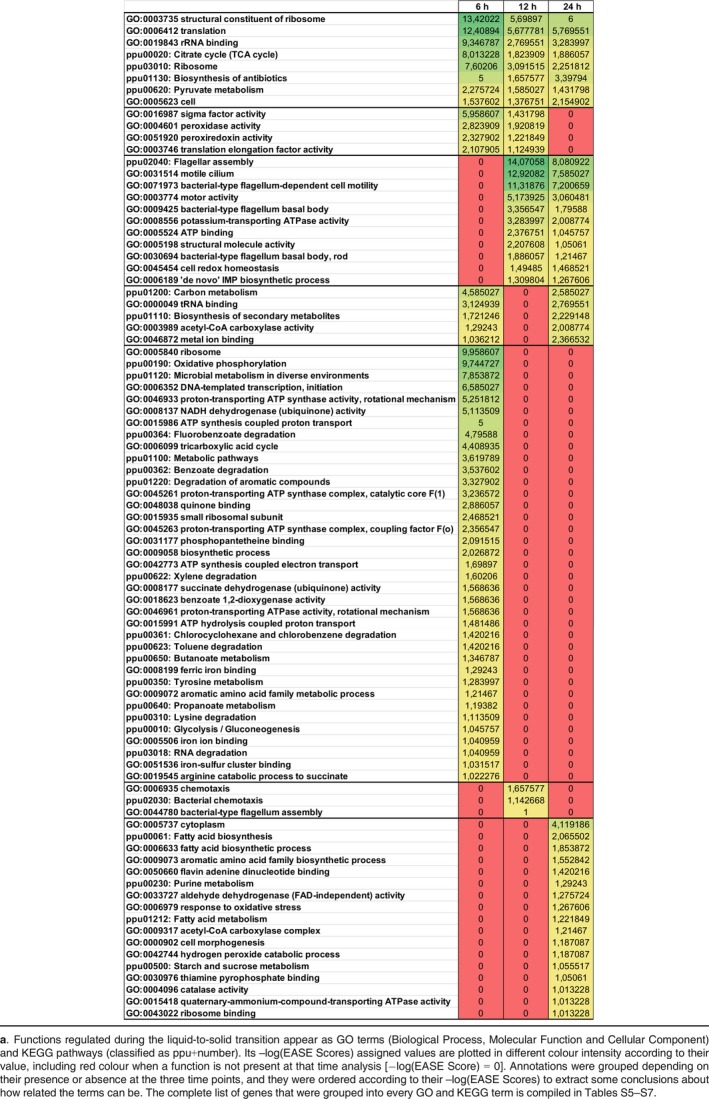
Heatmap‐style comparison^a^ of relevant functional motif groups in transcriptomic data from cells grown in liquid (reference) vs. solid (test) M9‐citrate medium at three time points

### Motility

One of the most affected genes in the transcriptomic comparisons was those involved in the regulation of flagellar activities which become of considerable importance at 12 and 24 h (Fig. 3, Table 1). Several GO and KEGG annotations can be noticed as relevant functions with a high –log(EASE Score), such as flagellar motility, motor activity, flagellar basal body or flagellar assembly. As a part of the flagellar motor, the stator portion is a very sensitive structure under sophisticated regulation that involves external factors such as viscosity or ion pressure, and also as a modulator of flagellar assembly and movement (Baker and O'Toole, 2017). If we take a closer look at the list of genes in Table 1 and Tables S2 and S3, just a few flagellar genes appear negatively regulated after 6 h of incubation (*flhB*,* fliJ*,* fliI*,* flgE*). But at 12 h, downregulation involves not only individual genes but also full operons (e.g. PP_4341 to PP_4394), and their fold‐change numbers are even lower at 24 h. In Table 1, we can also notice the onset of proteins with the ATP binding motifs, with a notable easy score at 12 and 24 h – perhaps related to flagellar functions also. Downregulation of some ORFs with homology to eukaryotic cilia (and thus likely to be involved in some type of motility) became noticeable at 12 and 24 h of incubation as well. Moreover, bacterial chemotactic activities became significantly undertranscribed at 12 h and after (PP_4332, PP_4888, PP_5020). Taken together, these observations strongly suggest that *Pseudomonas putida* stops the production of flagella, its assembly and the action of the rotor when cells are on solid, water‐unsaturated surfaces, presumably because they cannot swim and the maintenance of the machinery has a high metabolic cost (Martinez‐Garcia *et al*., 2014).

### Carbon metabolism

Central metabolism annotations such as the tricarboxylic acid (TCA) cycle and the intimately related oxidative phosphorylation are also overrepresented at 6 h of incubation according to the main GO terms and KEGG pathways appearing with low EASE Scores. It has to be noticed that *P. putida* KT2440 has a special metabolic network for central carbon metabolism that differs in some characteristics from the more studied *E. coli* pathways: although *P. putida* possesses most of the machinery for the most common Embden–Meyerhof–Parnas (EMP) glycolytic pathway, the gene *pfk* is missing from the genome and consequently the EMP pathway does not work as such. Instead, the EMP enzymes merge in a gluconeogenic direction with the Entner–Doudoroff (ED) pathway and the pentose phosphate (PP) pathway to create a distinct cycle that then links to the TCA cycle (Chavarria *et al*., 2013; Nikel *et al*., 2015; Sanchez‐Pascuala *et al*., 2017). On the other side, glyoxylate shunt activity is very low in this microorganism. Taken together, these singularities endow *P. putida* with a high level of reducing power and, therefore, a higher resistance to oxidative stress (Nikel *et al*., 2015).

Consistently with this, we found an upregulation of the ED, PP (upregulated *rpe*,* tktA*) and TCA pathway enzymes, specially in its reductive branch: *ipdG*,* sucB*,* sucA*, PP_2652 and PP_0897. This effect was accompanied by activation of pathways further from central carbon metabolism, including the oxidation of fatty acids. This suggested cells on solid medium have more energy requirements (at least at 6 h) than cultures in equivalent growth in liquid. We also noticed a positive regulation of EMP pathway (*tpiA*,* eno*), the glyoxylate shunt and the biosynthesis from C2 (at the level of pyruvate dehydrogenase activity by the enzymes *acoA*,* ace* and *ldpG*), indicating that intermediary metabolism is also more active in cells placed on solid medium (Arce‐Rodriguez *et al*., 2016; Reeves *et al*. 1996; Ramos *et al*., 2001). In sum, a general upregulation of carbon metabolism on agar plates becomes consistently apparent at all times (Table 1 and Tables S2–S4).

### Stress responses

Table 1 shows that peroxidase activity constitutes an important function at the earlier times (6 and 12 h). Later (24 h), the oxidative stress of solid cultures seems to demand less specific redox homeostasis functions. Taking a closer look at the list of results, we can also see a reorganization of the genes that act against oxidation over the three time points where the regulation of the activity of superoxide dismutases is more important at 6 h of incubation and the activity of catalases becomes more relevant at 12 h. This suggested a high presence of superoxide radicals at 6 h and a more prevalent action of hydrogen peroxide at later times at 12 h. Remarkably, the catalase gene *katA* increased its fold change from 45.36 at the 6 h comparison, to a dramatic 253.01 fold change at 12 h, and finally at 24 h of expression, *katA* becomes the highest overregulated gene of all, with a fold‐change value of 593.45.

The large overregulation of this group of oxidative stress‐related genes is due in part to the conditions of the solid media, for example higher level of desiccation and exposure to the metabolism of the neighbours in some other layers. But also, such a stress could be traced directly to being attached to a solid surface. This has been documented also in other cases. For example, some evidence relates oxidative stress with a higher production of c‐di‐GMP in *Klebsiella pneumoniae*. In this instance, the c‐di‐GMP phosphodiesterase YjcC is directly involved with the oxidation levels of the bacteria, as its transcription is controlled by the *soxRS* regulon (Huang *et al*., 2013). In *P. aeruginosa*, ROS and the oxidant agent hypochlorite (HClO) induce the expression of several diguanylate cyclases and biofilm formation as a possible protection strategy against oxygen radicals (Chua *et al*., 2016; Echeverz *et al*., 2017; Strempel *et al*., 2017), or in *E. coli*, the attachment ability can be induced with the stressor agent paraquat mediated by the PilZ domain protein DgcZ (Lacanna *et al*., 2016; Echeverz *et al*., 2017). The interplay between attachment, oxidation and c‐di‐GMP is intriguing and deserves further studies. Other stress‐related genes that become overexpressed in solid medium include PP_4707 (water subsaturation, unpublished), PP_4855, PP_1353, *cmpX* and some extracytoplasmic sigma factors.

### Other functions

Other genes and functions that appear in Table 1 become manifested at only one time point of the transcriptomic comparisons. For example, at 6 h, solid cultures show a drop of the metabolism of aromatic compounds and the transport of those molecules, but these functions are not differentially regulated at 12 or 24 h. We also found a downregulation of iron metabolism genes, as well as important transporters and transcription factors for these functions such as *pvdS*. Thus, the difference tends to disappear at 12 and 24 h, when the markers of iron metabolism tend to be the same under the two growth conditions. Similarly, pathways related to some amino acids such as lysine, tyrosine or arginine are divergent between solid and liquid medium at 6 h, but not at later times. It is remarkable that many components of the electron transport chain (e.g. the *nuo* genes) also become overexpressed at 6 h but not at 12 or 24 h. This could be related to the situation hinted at by the data above that bacteria need both more energy and extra reducing power for facing oxidative stress. In contrast, some chemotaxis activities peak at 12 h, whereas other functions related to the storage of carbon sources (e.g. fatty acid biosynthetic activities and starch metabolism) change by 24 h. We can even find genes encoding granule protein associated polyhydroxyalkanoates (PP_5007, PP_5008) in the results list, suggesting these ways of C saving become important. Other activities that pop up at 24 h include biosynthesis of aromatic amino acids, further responses to oxidative stress, ammonium transport, cytoplasmic activities and cell morphogenesis.

Further inspection of the gene/function lists of Table 1 and Tables S2–S4 beyond the classification with DAVID allows identification of upregulated genes of the type 6 secretion systems (T6SSs). T6SSs have been described as molecular devices that perform different activities, in particular of toxins to eukaryotic or (mostly) prokaryotic neighbours, biofilm formation and regulation of some genes (Silverman *et al*., 2011). Among our data, we find increased transcription of *hcp1* (PP_3089) and other components of T6SS cluster 1 (from PP_3088 to PP_3100) at 6 h. Nevertheless, just a few components of T6SS cluster 3, such as *tssM3* (PP_2627), are upregulated at 12 h of incubation.

Surprisingly, very few of the 43 proteins annotated in *P. putida*'s genome as containing diguanylate cyclase/phosphodiesterase domains, appeared in our analysis with either negative or positive fold‐change numbers. The molecule produced or degraded by them, c‐di‐GMP, is the main secondary messenger controlling attachment to solid surfaces (Römling *et al*., 2013). This is not exclusive of *P. putida*, as the same set of genes vary also little in *Pseudomonas fluorescens* growing in solid vs. liquid media (Dahlstrom *et al*., 2018). It is plausible that the major regulatory layer of these proteins is post‐translational (Dahlstrom *et al*., 2015, 2018), and thus, their transcriptional regulation may not be that critical.

## Conclusion

The protocols and the study case presented above exemplify a complete workflow for inspecting the major physiological differences that *P. putida* undergoes when grown on two different conditions – in this specific case, the surface of agar in a Petri dish vs. growth in a liquid medium. The most conspicuous dissimilarities include a much higher translational and metabolic activity when growing on the surface (at ~6 hrs) and the inhibition of flagellar genes (and thus reduced motility). Moreover, cells grown on a surface present also multiple indications of being subject to a strong oxidative stress, perhaps related to direct exposure of their biomass to the oxygen in air. These are general trends that are accredited by the large number of functional genes that consistently vary under the circumstances. However, studies on the role of specific genes need to be verified (e.g. by QT‐PCR) and followed up on a case‐by‐case basis, an endeavour that is beyond the scope of this protocol article.

The herein described methodology is generally applicable to any other situation where the consequences of environmental shifts between two or more conditions need to be assessed. Although the methods are optimized for *P. putida* strain KT2440, they should be generally applicable to other *Pseudomonas* strains and other Gram‐negative bacteria in general. While the computational platforms for transcriptomic data analysis will surely improve from time to time, the reliability of the results will always rely to a large extent on the quality of RNA preparations.

## Conflict of interest

None declared.

## Supporting information


**Table S1**. Primers used for DNA detection in RNA samples by qRT‐PCR.
**Table S2**. List of down and upregulated genes comparing *P. putida* KT2440 cultures in solid vs. liquid media at 6 h of incubation.
**Table S3**. List of down and upregulated genes comparing *P. putida* KT2440 cultures in solid vs liquid media at 12 h of incubation.
**Table S4**. List of down and upregulated genes comparing *P. putida* KT2440 cultures in solid vs liquid media at 24 h of incubation.
**Table S5**. DAVID Functional analysis results for 6 h comparison.
**Table S6**. DAVID functional analysis results for 12 h comparison.
**Table S7**. DAVID functional analysis results for 24 h comparison.

## Note

1 Buffer RLT contains a high concentration of guanidine isothiocyanate, which supports the binding of RNA to the silica membrane. But, alas, its exact composition is confidential as a proprietary component of Qiagen RNeasy Kits.
